# Oestrogen and androgen receptors in melanoma.

**DOI:** 10.1038/bjc.1980.112

**Published:** 1980-04

**Authors:** P. Rümke, J. P. Persijn, C. B. Korsten


*Br. J. Cancer* (1980) **41**, 652

***Short Communication***

### OESTROGEN AND ANDROGEN RECEPTORS IN MELANOMA

P. RÜMKE, J. P. PERSIJN AND C. B. KORSTEN

*From the Department of Internal Medicine and Department of Clinical Chemistry, The Netherlands Cancer Institute, Amsterdam, The Netherlands*

Received 20 June 1979 Accepted 10 December 1979

GROWTH OF MALIGNANT MELANOMA has occasionally been shown to be hormone-dependent. Bodenham & Hale (1972) administered $^{32}P$ to patients with metastases of malignant melanoma and measured the uptake in the metastases and in control tissues with a Geiger probe. After a few days, when the uptake curve was found to be constant, oestrogens were administered. A change in the uptake curve was found in 4/26 cases. Two of these 4 patients were hypophysectomized and showed remissions for at least 9 months. The 2 others showed regression after administration of either testosterone phenylpropionate in a man and ethinyl oestradiol in a woman. In another report on 5 patients, a melanoma developed or became worse after oestrogen administration (Sadoff *et al.*, 1973). In a preliminary study regression was reported in 5/44 melanoma patients after treatment with the anti-oestrogenic progestational drug 6α - methylpregn - 4 - ene - 3,11,20 - trione (NSC-17256; Johnson *et al.*, 1966). Several authors have reported the influence of pregnancy on the course of melanoma. References can be found in the paper of Shiu *et al.* (1976) who showed in their study that with Stage II but not with Stage I melanoma patients the rate of survival for 5 years free of disease was significantly lower in patients who were treated during pregnancy or who had symptoms of activation of the skin lesion during a previous pregnancy, than in those who were nulliparous or who had no symptoms of activation during a previous pregnancy. In contrast to an adverse effect of pregnancy stands the remarkable case of a patient whose melanoma regressed during successive pregnancies (Boyd, 1957). Recently Shaw *et al.* (1978) concluded from a study on a large series of patients that there may be endocrine influences on the rate of formation of metastases and the anatomical distribution of the sites of primary lesions.

Although hormonal dependency of the growth rate of metastases has clearly been shown, it occurs in so few patients that hormonal treatment of melanoma patients with advanced disease, as is practised in mammary carcinoma, is not customary. However, endocrine therapy would become the treatment of choice if it were possible to select appropriate patients by some criterion for hormone dependency. This situation is possible in breast-cancer patients where specific binding of oestrogens to receptor in tumour extract has been shown to correlate with successful hormonal treatment (McGuire *et al.*, 1975). So far, only Fisher *et al.* (1976) have reported the detection of oestrogen receptor activity in the metastases of 16/35 patients with malignant melanoma.

It is the aim of this paper to report on the binding activity to oestrogen and dihydro-testosterone by melanoma metastases, as found with the routine procedures which are in use in our laboratory for the detection of hormone-receptor activity in breast cancer.

*Hormone-receptor assay*

Extracts of the tumour tissues were prepared as recommended at the workshop in 1972 of the EORTC Breast Cancer Cooperative Group (1973), using a "microdismembrator" (Braun, Melsungen, Germany). Incubation with the labelled oestrogen [(6,7-$^3$H) oestradiol-17$\beta$] ($E_2$), 40 Ci/mmol or androgen [5-dihydro (1,2-$^3$H)-testosterone] (DHT), 48 Ci/mmol was done at 4°C for about 16 h. The final concentration of the labelled hormone was 3 nM. Total protein was assayed by the biuret reaction. Human serum albumin was assayed immunologically by single diffusion using Partigen plates, manufactured by Behring Werke (Germany). The estimated amount of serum proteins in the cytosol was calculated by multiplying the albumin concentration by a factor of 10/6 (assuming an albumin concentration of 60% in the extracellular protein contaminants of the extracts). The amount of soluble tissue proteins is then the total protein amount minus the amount of serum proteins. Oestrogen and androgen receptors were determined in the extract using agar electrophoresis. This technique has been described in detail by Wagner (1972). The electrophoresis equipment used in our study was an exact replica of the one described in the paper of Wagner. The electrophoresis separates high-affinity receptors from sex-hormone binding to globulin and unbound hormone, since the former migrates to the anode while the sex-hormone binding globulin and unbound hormone are shifted towards the cathode. As a control we used heated cytosol (1 h at 45°C). The difference between the anodal peaks of the unheated and preheated cytosol was used as a measure of high-affinity steroid binding capacity, which was expressed in fmol of receptor bound per mg tissue protein.

*Metastatic excisions*

$E_2$ and DHT receptors were determined in excised metastases of patients with histologically proven disseminated malignant melanoma. All specimens were frozen within 1 h and stored at −70°C before the extracts were made. Thirty-nine extracts were assayed within 12 days, and 4 within 4 months. All assays were performed simultaneously with extracts of breast cancers.

The Table shows the results of the assays on 21 metastases of 17 male and 22 metastases of 17 female patients. $E_2$ receptor was measurable in 7/31 cutaneous and 1/9 lymphnode metastases, the concentration in this lymphnode metastasis being the lowest of all measurable assays. In the 3 non-skin, non-lymphnode metastases no $E_2$ receptor was detectable. There is no relationship between the sex of the patients and the presence of $E_2$ receptor in a skin metastasis, and no relationship between the presence of detectable $E_2$ and DHT receptor. DHT-binding activity was found in 7/31 cutaneous, 4/9 lymphnode metastases, and 0/3 non-skin, non-lymphnode metastases. In one patient there was DHT activity found in a skin and a lymphnode metastasis excised at the same time. In 6 patients both assays were performed on another metastasis at another time. In 3 cases the first but not the second, and in 2 cases the second but not the first had detectable $E_2$ receptors. In 2 cases DHT receptor was detectable, one the first and one the second time. Thus, in no case was a receptor detectable on both occasions.

The 2 women with the highest receptor activity (one with $E_2$, the other with DHT) had never been pregnant, nor had they used oral contraceptives. Nearly all patients in whom receptor activity was found had progressive disease; only one man (76/1909) with DHT binding activity in a removed regional lymphnode metastasis, is still disease-free 3 years later. No relationship was found between the presence of receptor and the prognosis of the disease or the age or sex of the patient.

In the 4 instances in which the specimens had to be stored for 4 months, there were 2 cases where $E_2$ receptor was detectable (1·1 and 11·6 fmol/mg).

TABLE

| Patient No. | Sex | Age | Site of excision | Date of excision | Binding of $E_2$ (fmol/mg cytosol tissue protein) | Binding of DHT (fmol/mg cytosol tissue protein) |
|---|---|---|---|---|---|---|
| 73/0432 | M | 57 | Skin | 05.07.73 | —* | —* |
| 73/0432 | | | Skin | 05.07.73 | — | — |
| To. (LDI) | M | 41 | Skin | 28.12.73 | — | 0·30 |
| To. (LDI) | | | Lymph node | 28.12.73 | — | 0·98 |
| 72/1398 | M | 57 | Jejunum | 07.02.74 | — | — |
| 71/0288 | M | 28 | Skin | 08.04.74 | — | 13 |
| 74/0813 | M | 61 | Skin | 24.04.75 | — | 5·5 |
| 74/0813 | | 62 | Lymph node | 09.10.75 | — | — |
| 75/0533 | M | 91 | Skin | 24.04.75 | — | — |
| 72/2032 | M | 76 | Skin | 02.06.75 | — | — |
| 75/1413 | M | 48 | Skin | 19.06.75 | — | — |
| 75/1037 | M | 44 | Lymph node | 08.07.75 | — | — |
| 72/2142 | M | 33 | Skin | 17.11.75 | 8·0 | — |
| 72/2142 | | 34 | Skin | 01.12.76 | — | — |
| 74/2413 | M | 49 | Skin | 03.09.75 | — | — |
| 75/2903 | M | 38 | Skin | 17.12.75 | — | — |
| 75/1875 | M | 24 | Skin | 19.12.75 | 3·8 | 11·5 |
| 74/2213 | M | 52 | Skin | 31.12.75 | — | 0·38 |
| 76/0124 | M | 47 | Skin | 23.01.76 | 6·9 | — |
| 76/0142 | M | 54 | Lymph node | 04.02.76 | — | — |
| 76/1909 | M | 57 | Lymph node | 15.09.76 | — | 16·7 |
| 73/1300 | F | 65 | Skin | 20.07.73 | — | — |
| 73/1300 | | 66 | Skin | 14.03.74 | 1·1 | — |
| 70/0267 | F | 43 | Skin | 23.07.73 | 22 | — |
| 70/0267 | | 45 | Skin | 17.06.75 | — | — |
| 73/2138 | F | 84 | Lymph node | 30.11.73 | — | 0·22 |
| 74/0385 | F | 28 | Skin | 20.02.74 | 11·6 | — |
| 73/0186 | F | 62 | Lymph node | 27.02.74 | — | — |
| 75/0191 | F | 27 | Breast | 13.02.75 | — | — |
| 74/2087 | F | 27 | Lymph node | 18.04.75 | 0·6 | — |
| 74/2087 | | 28 | Skin | 12.08.76 | — | — |
| 73/1469 | F | 37 | Skin | 08.07.75 | — | — |
| 73/0959 | F | 73 | Skin | 17.07.75 | — | — |
| 69/0602 | F | 62 | Skin | 05.08.75 | — | 22 |
| 73/1773 | F | 38 | Skin | 17.09.75 | — | — |
| 75/2558 | F | 70 | Lymph node | 20.11.75 | — | 1·8 |
| 75/1809 | F | 33 | Skin | 17.12.75 | — | — |
| 75/1809 | | 33 | Skin | 05.03.76 | 8·9 | 0·72 |
| 76/0549 | F | 27 | Skin | 12.03.76 | — | — |
| 76/0549 | | | Skin | 12.03.76 | — | — |
| 72/1880 | F | 56 | Skin | 28.07.76 | — | — |
| 73/2155 | F | 39 | Lung | 24.03.76 | — | — |
| 75/0937 | F | 53 | Skin | 01.11.76 | — | — |

* Not measurable ($< 0{\cdot}5$ fmol $E_2$ and $< 0{\cdot}15$ fmol DHT/mg cytosol tissue protein).

Two male patients (71/0288 and 75/1875) received hormonal treatment after a high DHT-binding activity had been found. The first man, 28 years old, received 3 mg ethinyloestradiol daily for 6 weeks, and thereafter the antitestosterone drug cyproterone acetate, 100 mg daily, but without any effect on the progression of the lung and skin metastases. The other patient, 24 years old, only received cyproterone acetate in the last 2 weeks of his life, without any effect on the progression of the disease.

This study shows that $E_2$ and DHT receptors can be present in some melanoma metastases. According to studies on breast cancer, $E_2$-binding activity of more than 15–30 fmol/mg cytosol tissue protein is considered as "receptor-positive". According to this criterion we found only one female patient having $E_2$ receptor on at least one occasion in a skin metastasis. Although detectable in 20–25% of the patients the levels are generally too low to be considered of any relevance to endocrine treatment. Moreover, in 6 cases (2 males and 4 females) there was no consistently detectable receptor present, while in none of them was receptor present on 2 occasions.

The incidence of $E_2$ positivity in our study is lower than reported by Fisher *et al.* (1976) who found 16/35 patients with levels higher than 5 fmol/mg cytosol. Another difference from their findings concerns the relationship with the sites of the metastases. While we found $E_2$ receptors virtually only in skin (7/31) and hardly in lymphnode metastases (1/9), Fisher *et al.* (1976) found a prevalence for lymphnode metastases (9/18 of lymph node and 4/12 of skin metastases). In their study, however, a different technique, including the reagent dithiothreitol (DTT) and analysis of the results by Scatchard plot was used. DTT has been suggested to increase the yield of the receptor assay (McGuire & DeLaGarza, 1973; Ratajczak & Hähnel, 1976), but other laboratories (Mester *et al.*, 1970; Braunsberg, 1975; Keightley *et al.*, 1978) including ours (unpublished results) have found that this reagent is not effective. The analysis by Scatchard plot may lead to erroneous results in the assay of cytosols where the contamination with plasma-binding proteins of lower specificity may be considerable. A critical discussion of this problem from which misclassification of receptors or miscalculation of the dissociation constant ($K_D$) may result, is given by Braunsberg (1975). For the present discussion we would remark that in the quoted study of Fisher *et al.* (1976) a constant ($K_D$) as high as $4{\cdot}9 \times 10^{-9}$M was calculated, which was considered to represent high-affinity binding. Such a high value, however, may, according to Braunsberg (1975) and others (*vide* McGuire *et al.*, 1975) rather suggest a low-affinity binding. This could then explain the higher incidence of $E_2$ receptors in melanoma in the study of Fisher *et al.* (1976) when compared to our virtually negative results. In our study no $K_D$ values could be determined, since the technique used is based on incubation with a single saturating oestradiol concentration. There are, however, several arguments that sex-hormone-binding globulin did not interfere in our assay for the receptors. The electrophoresis technique is the only procedure currently available which separates distinctly specific receptor proteins and sex-hormone-binding globulins, in fact the method allows the separate determination of sex-hormone-binding globulin (Wagner & Rüffert, 1974). Our electrophoretic diagrams obtained with the Wagner technique conformed to those published by various investigators (Wagner, 1972; Krieg *et al.*, 1974; Wagner & Jungblut, 1976*a,b*; Trams & Maass, 1977). Moreover, the correlation between the results obtained with the electrophoretic method and the charcoal method for oestrogen receptor is excellent (Korsten & Persijn, 1977).

In 11 cases there was measurable DHT-binding activity. The higher levels above 5 fmol/mg tissue cytosol were found in 4 men and one woman, the latter having the highest level. Also DHT binding activity was not related to the state of the disease, nor to the age and sex of the patients. Two young male patients with advanced disease received anti-testosterone treatment, and one also received ethinyl oestradiol in high doses. However, these treatments had no effect on the progression of the disease. The dose of the anti-androgens may have been too low or the treatment too short, but it may also be that the fraction of melanoma cells without receptor was too large, and it may also be that proliferation of the cells in spite of the presence of a receptor does not require the stimulus of the appropriate hormones. Recently also, Fisher *et al.* (1978) showed that the presence of $E_2$ receptor did not correlate with the response on the administration of diethylstilboestrol, which occurred only in 2 patients, both having no

$E_2$-binding activity, while 4/18 patients with $E_2$ receptor in metastases did not respond.

It can therefore tentatively be concluded that receptor determinations are of no help in the management of patients with advanced malignant melanoma.

The authors are indebted to Dr E. P. van der Esch for the pathological examination of the tumours.

The skilful assistance of Mrs A. C. M. Brakeboer and Mrs M. Verzijde is gratefully acknowledged.

This work was supported by a grant from the Maurits and Anna de Kock Fund.

### REFERENCES

Bodenham, D. C. & Hale, B. (1972) Malignant melanoma. In *Endocrine Therapy in Malignant Disease*. Ed. B. A. Stoll. London: Saunders. p. 377.

Boyd, W. (1957) Spontaneous regression of cancer. *Can. Ass. Radiol.*, **8**, 45.

Braunsberg, H. (1975) Factors influencing the estimation of oestrogen receptors in human malignant breast tumours. *Eur. J. Cancer*, **11**, 499.

EORTC Breast Cancer Cooperative Group (1973) Standards for the assessment of estrogen receptors in human breast cancer. Report of a workshop. *Eur. J. Cancer*, **9**, 379.

Fisher, R. I., Neifeld, J. P. & Lippman, M. E. (1976) Oestrogen receptors in human malignant melanoma. *Lancet*, ii, 337.

Fisher, R. I., Young, R. C. & Lippman, M. E. (1978) Diethylstilbestrol therapy of surgically non-resectable malignant melanoma. *Proc. Am. Ass. Cancer Res.*, **19**, 139.

Johnson, R. D., Bisel, H., Andrews, N. & 6 others (1966) Phase I clinical study of 6 methyl-pregn-4-ene-3,11,20, trione (NSC 17256). *Cancer Chemother. Rep.*, **50**, 571.

Keightley, D. D., Tilley, W. D. & Cant, E. L. M. (1978) Effect of snap freezing, dithiothreitol and storage on estimations of estrogen receptor sites. *Clin. Chim. Acta*, **88**, 337.

Korsten, C. B. & Persijn, J. P. (1977) Evaluation of and additional data on an improved simple charcoal method to determine oestrogen receptors. *J. Clin. Chem. Clin. Biochem.*, **15**, 297.

Krieg, M., Steins, P., Szalay, R. & Voigt, K. D. (1974) Characterization of a specific androgen receptor in rat prostate cytosol by agargel electrophoresis. *In vivo* and *in vitro* studies. *J. Steroid Biochem.*, **5**, 87.

McGuire, W. L. & DeLaGarza, M. (1973) Similarity of the estrogen receptor in human and rat mammary carcinoma. *J. Clin. Endocrinol. Metab.*, **36**, 548.

McGuire, W. L., Carbone, P. P. & Vollmer, E. P. (1975) *Estrogen Receptors in Human Breast Cancer*. New York: Raven Press.

Mester, J., Robertson, D. M., Feherty, P. & Kellie, A. E. (1970) Determination of high-affinity oestrogen receptor sites in uterine supernatant preparations. *Biochem. J.*, **120**, 831.

Ratajczak, T. & Hähnel, R. (1976) Estradiol receptors: influence of plasma proteins on detection and quantitation. *J. Steroid Biochem.*, **7**, 741.

Sadoff, L., Winkley, J. & Tyson, S. (1973) Is malignant melanoma an endocrine-dependent tumor? The possible adverse effect of estrogen. *Oncology*, **27**, 244.

Shaw, H. M., Milton, G. W., Farago, G. & McCarthy, W. H. (1978) Endocrine influences on survival from malignant melanoma. *Cancer*, **42**, 669.

Shiu, M. H., Schottenfeld, D., Maclean, B. & Fortner, J. G. (1976) Adverse effect of pregnancy on melanoma. *Cancer*, **37**, 181.

Trams, G. & Maass, H. (1977) Specific binding of estradiol and dihydrotestosterone in human mammary cancers. *Cancer Res.*, **37**, 258.

Wagner, R. K. (1972) Characterization and assay of steroid hormone receptors and steroid-binding serum proteins by agargel electrophoresis at low temperature. *Hoppe-Seyler's Z. Physiol. Chem.*, **353**, 1235.

Wagner, R. K. & Jungblut, P. W. (1976*a*) Differentiation between steroid hormone receptors CBG and SHBG in human target organ extracts by a single-step assay. *Mol. Cell. Endocrinol.*, **4**, 13.

Wagner, R. K. & Jungblut, P. W. (1976*b*) Oestradiol and dihydrotestosterone receptors in normal and neoplastic human mammary tissue. *Acta Endocrinol.*, **82**, 105.

Wagner, R. K. & Rüffert, W. (1974) Assay of CBG and SHBG by agargel electrophoresis. *Acta Endocrinol. Suppl.*, **184**, 14.


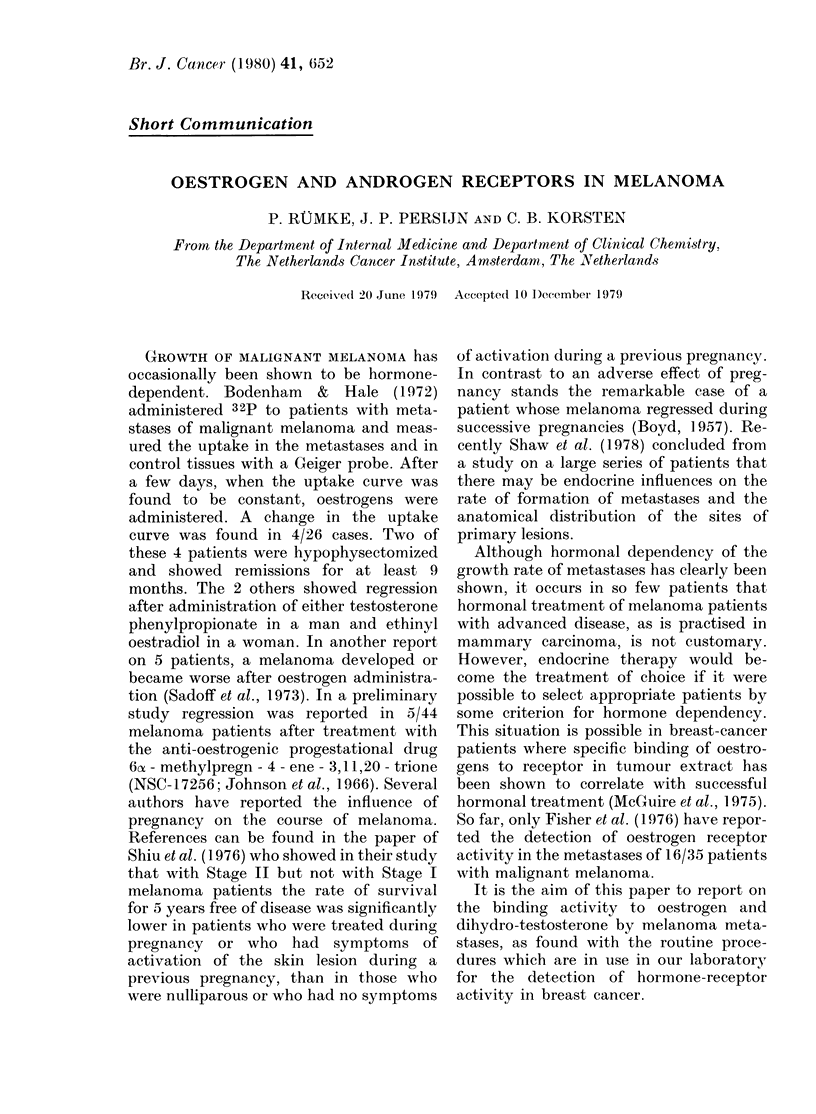

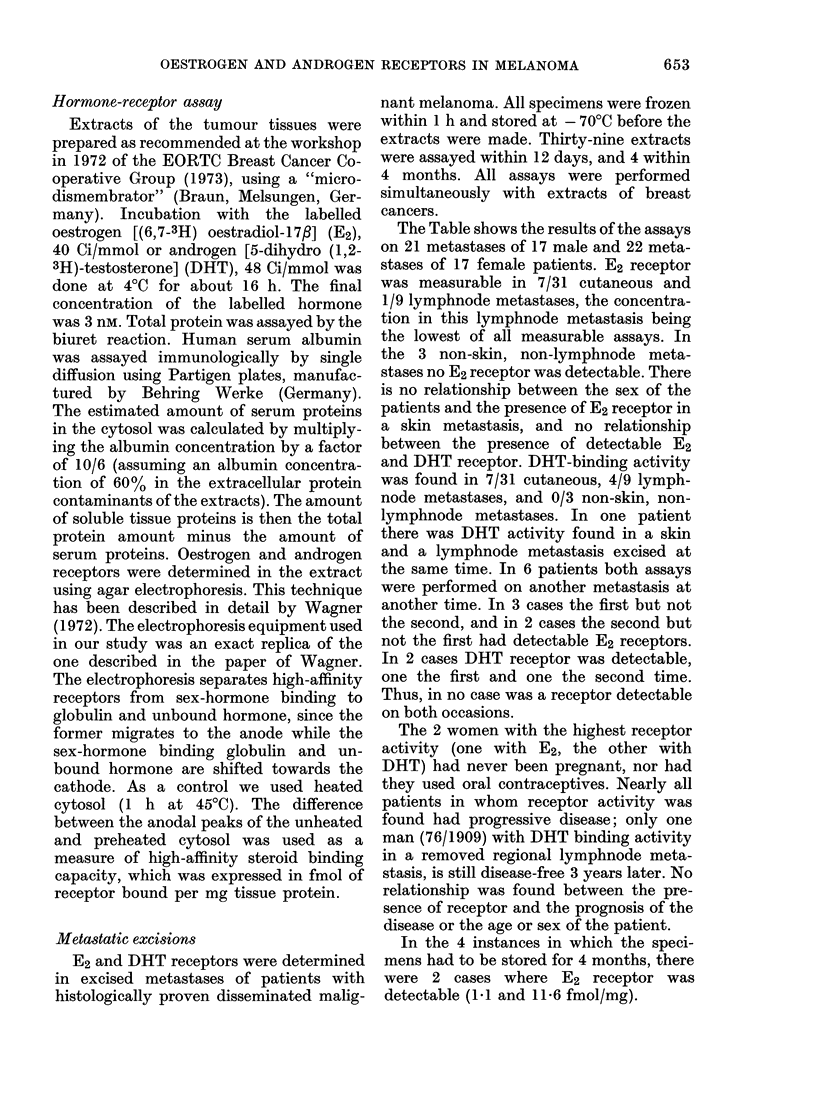

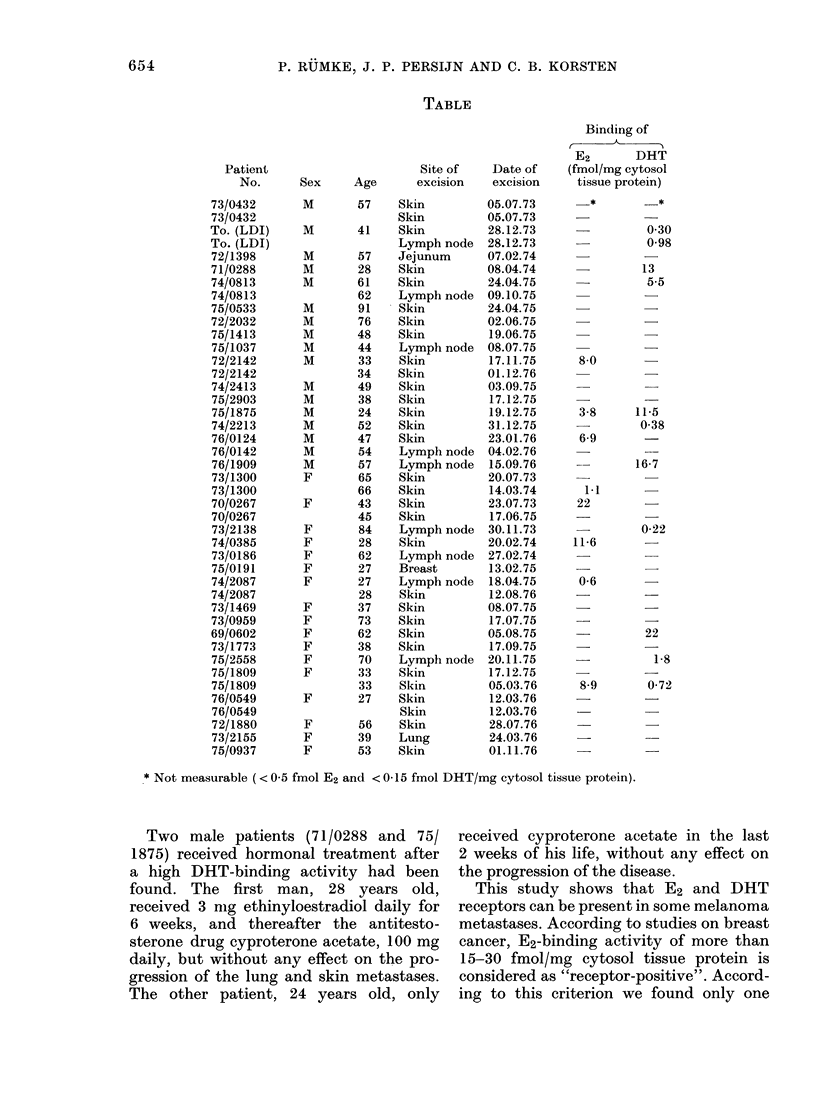

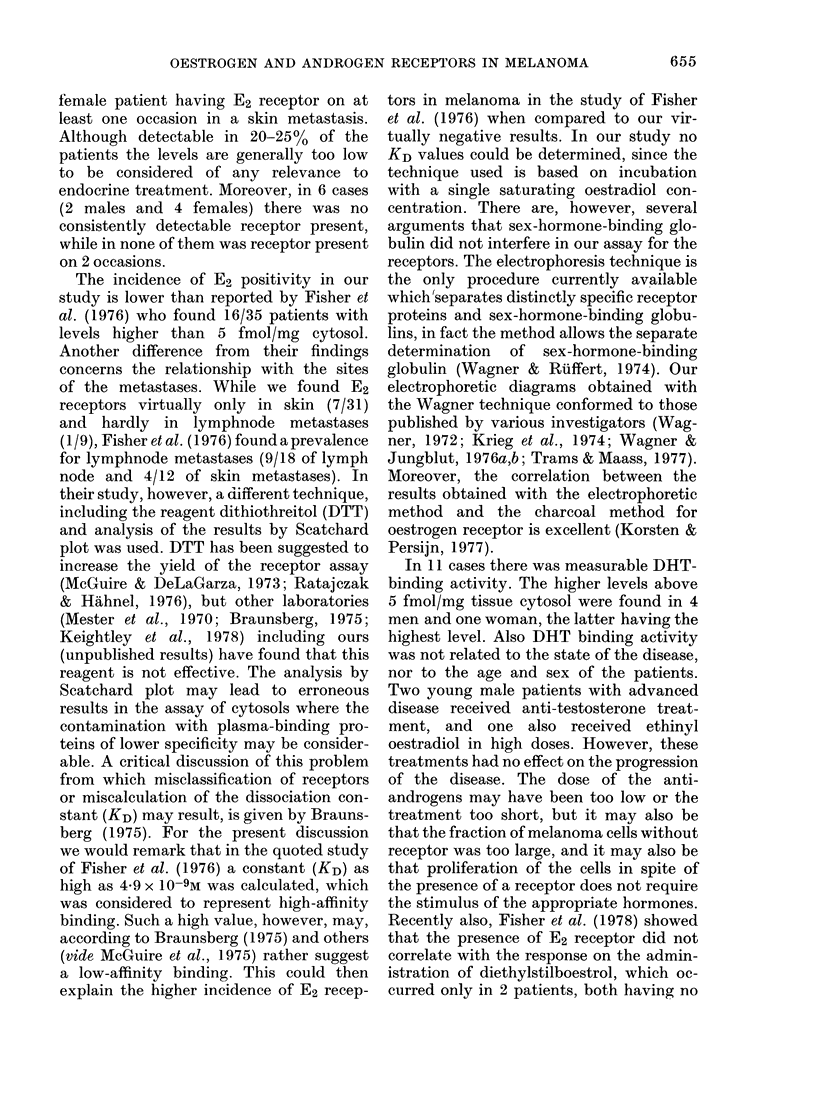

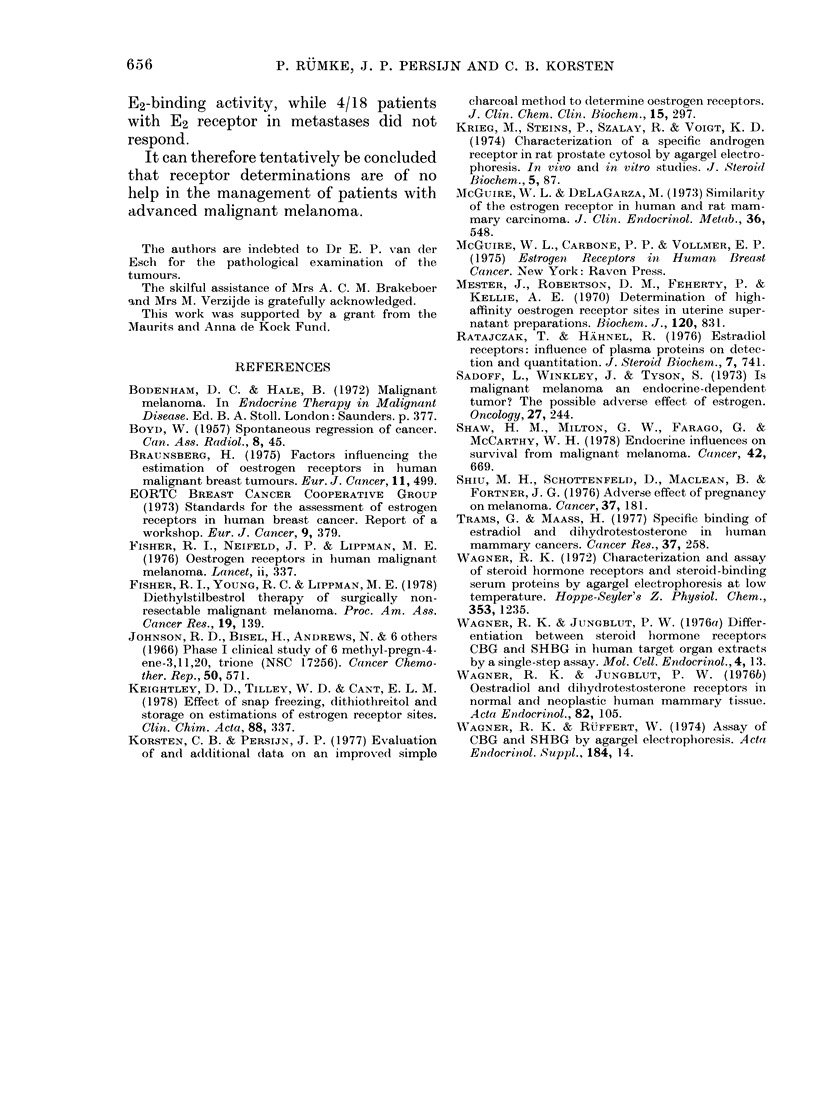


## References

[OCR_00593] Braunsberg H. (1975). Factors influencing the estimation of oestrogen receptors in human malignant breast tumours.. Eur J Cancer.

[OCR_00603] Fisher R. I., Neifeld J. P., Lippman M. E. (1976). Oestrogen receptors in human malignant melanoma.. Lancet.

[OCR_00620] Keightley D. D., Tilley W. D., Cant E. L. (1978). Effect of snap freezing, dithiothreitol and storage on estimations of estrogen receptor sites.. Clin Chim Acta.

[OCR_00626] Korsten C. B., Persijn J. P. (1977). Evaluation of and additional data on an improved simple charcoal method to determine oestrogen receptors.. J Clin Chem Clin Biochem.

[OCR_00633] Krieg M., Steins P., Szalay R., Voigt K. D. (1974). Characterization of a specific androgen receptor in rat prostate cytosol by agargel electrophoresis. In vivo and in vitro studies.. J Steroid Biochem.

[OCR_00640] McGuire W. L., DeLaGarza M. (1973). Similarity of the estrogen receptor in human and rat mammary carcinoma.. J Clin Endocrinol Metab.

[OCR_00651] Méster J., Rbertson D. M., Feherty P., Kellie A. E. (1970). Determination of high-affinity oestrogen receptor sites in uterine supernatant preparations.. Biochem J.

[OCR_00657] Ratajczak T., Hähnel R. (1976). Estradiol receptors: influence of plasma proteins on detection and quantitation.. J Steroid Biochem.

[OCR_00661] Sadoff L., Winkley J., Tyson S. (1973). Is malignant melanoma an endocrine-dependent tumor? The possible adverse effect of estrogen.. Oncology.

[OCR_00667] Shaw H. M., Milton G. W., Farago G., McCarthy W. H. (1978). Endocrine influences on survival from malignant melanoma.. Cancer.

[OCR_00673] Shiu M. H., Schottenfeld D., Maclean B., Fortner J. G. (1976). Adverse effect of pregnancy on melanoma: a reappraisal.. Cancer.

[OCR_00678] Trams G., Maass H. (1977). Specific binding of estradiol and dihydrotestosterone in human mammary cancers.. Cancer Res.

[OCR_00683] Wagner R. K. (1972). Characterization and assay of steroid hormone receptors and steroid-binding serum proteins by agargel electrophoresis at low temperature.. Hoppe Seylers Z Physiol Chem.

[OCR_00690] Wagner R. K., Jungblut P. W. (1975). Differentiation between steroid hormone receptors CBG and SHBG in human target organ extracts by a single-step assay.. Mol Cell Endocrinol.

[OCR_00695] Wagner R. K., Jungblut P. W. (1976). Oestradiol- and dihydrotestosterone receptors in normal and neoplastic human mammary tissue.. Acta Endocrinol (Copenh).

